# A novel machine learning model for predicting clinical pregnancy after laparoscopic tubal anastomosis

**DOI:** 10.1186/s12884-023-05854-5

**Published:** 2023-07-24

**Authors:** Nan Ding, Jian Zhang, Peili Wang, Fang Wang

**Affiliations:** Reproductive Medicine Center, Lanzhou University Second Hospital, No.82, Cuiying Road, Chengguan District, Lanzhou City, Gansu Province China

**Keywords:** Machine learning, LASSO regression, Laparoscopic tubal anastomosis, Prediction model

## Abstract

**Background:**

Laparoscopic tubal anastomosis (LTA) is a treatment for women who require reproduction after ligation, and there are no reliable prediction models or clinically useful tools for predicting clinical pregnancy in women who receive this procedure. The prediction model we developed aims to predict the individual probability of clinical pregnancy in women after receiving LTA.

**Methods:**

Retrospective analysis of clinical data of patients undergoing LAT in the Second Hospital of Lanzhou University from July 2017 to December 2021. Least absolute shrinkage and selection operator (LASSO) regression was used for data dimension reduction and feature selection. We incorporated the patients’ basic characteristics, preoperative laboratory tests and laparoscopic tubal anastomosis procedure signature and obtained a nomogram. The model performance was evaluated in terms of its calibration, discrimination, and clinical applicability. The prediction model was further internally validated using 200 bootstrap resamplings.

**Results:**

A total of 95 patients were selected to build the predictive model for clinical pregnancy after LTA. The LASSO method identified age, intrauterine polyps, pelvic adhesion and thyroid stimulating hormone(TSH) as independent predictors of the clinical pregnancy rate. The prediction nomogram included the abovementioned four predictive parameters. The model showed good discrimination with an area under the curve (AUC) value of 0.752. The Hosmer‒Lemeshow test of calibration showed that χ2 was 4.955 and the p value was 0.838, which indicates a satisfactory goodness-of-fit. Decision curve analysis demonstrated that the nomogram was clinically useful. Internal validation shows that the predictive model performs well.

**Conclusion:**

This study presents a nomogram incorporating age, intrauterine polyps, pelvic adhesion and TSH based on the LASSO regression model, which can be conveniently used to facilitate the individualized prediction of clinical pregnancy in women after LTA.

## Introduction

Although there are several options for postpartum contraception [1], tubal ligation remains one of the conventional methods among women who have already given birth to two children. Many reasons, such as the loss of children or divorce followed by remarriage, may provide women who have undergone tubal ligation the desire to have additional children [2]. For these patients, an alternate therapeutic option is either surgery or in vitro fertilization-embryo transfer(IVF-ET) [3, 4]. However, IVF-ET has some potential complications or risks, including multiple pregnancies and ovarian hyperstimulation syndrome [5, 6]. Tubal anastomosis is regarded as a cost-effective operation to reconstruct the structure and function of the fallopian tubes and is the only way for patients to conceive naturally after tubal ligation [7]. Successful tubal anastomosis allows natural conception in each ovulation cycle. For patients with a history of tubal sterilization, most are of advanced maternal age, and the optimal reproductive age is missed, so there is an urgent need for patients to know the probability of pregnancy after undergoing LTA. For physicians, it is also necessary to know the postoperative pregnancy rate to provide patients with more individualized and effective pregnancy treatment protocols.

Currently, there is no good prediction model for predicting the probability of pregnancy after LTA. Some patients miss the optimal period for IVF and waste considerable time and medical resources. Therefore, an effective prediction model is needed to predict and manage those patients to facilitate more individualized clinical decisions. Machine learning has been increasingly used in medical practice due to its potential to improve clinical decision-making and patient outcomes. The application of machine learning methods has improved artificial intelligence and has been used in clinical prediction [8–10]. LASSO regression is a machine learning technique for linear regression that employs L1 regularization to build models and select variables. By penalizing the model coefficients, L1 regularization shrinks certain coefficients to zero, thereby facilitating variable selection. The key advantage of Lasso regression is its ability to efficiently choose variables, reduce model complexity, and prevent overfitting [11]. This method is particularly effective in analyzing high-dimensional data and can autonomously determine the best set of features, reducing the need for manual selection [12]. In this study, we propose a LASSO regression model that takes advantage of patients’ baseline clinical parameters, preoperative laboratory tests, and intraoperative pelvic conditions to predict postoperative pregnancy rates.

## Methods

This retrospective study was conducted at Lanzhou University Second Hospital Reproductive Medicine Centre. All patients refused IVF-ET and signed informed consent before LTA, and the study conformed to the Declaration of Helsinki. The study was approved by the Lanzhou University Second Hospital Ethics Committee (No: 2022 A-439).

### Patient selection

Between July 2017 and December 2021, patients (aged between 19 and 47 years) who underwent LTA were collected. All participants underwent preoperative assessments, including blood tests, biochemistry, coagulation, infectious diseases, and fertility evaluations, including an assessment of their menstrual status and the number of follicles displayed on ultrasound. The exclusion criteria were as follows: (1) Either partner suffers from illnesses that are not suitable for pregnancy, such as severe heart disease, high blood pressure, serious kidney or liver disease, etc.; (2) During the operation, we found that the bilateral fallopian tubes had no normal anatomical structure and could not be treated surgically; (3) Male partners with abnormal semen analysis, such as azoospermia or high levels of malformed sperm.

Finally, a total of 126 eligible individuals who underwent tubal anastomosis were collected. Among these, 28 patients without follow-up and 3 patients with ectopic pregnancies were excluded. Finally, 95 eligible patients were selected in the study to build the prediction model.

### Data collection

All participants’ personal information was used anonymously, and clinical parameters were incorporated into and evaluated in the model: (1) Patients’ basic characteristics, including age, body mass index(BMI), race, education, and sterilization duration. (2) Preoperative laboratory tests included free triiodothyronine (FT3), free thyroxine(FT4), TSH, cancer antigen 125(CA125), homocysteine(HCY), D-dimer and antral follicle count(AFC). (3) Documentation of LTA surgical procedure, including anastomosis site, and various complications such as hydrosalpinx, hysteromyoma, ovarian cyst, pelvic adhesion, intrauterine polyps, endometriosis. All the above data were extracted from the medical records.

### Statistical analysis

Continuous variables are presented as the mean ± SD or median (25th–75th percentiles) as appropriate. Differences between groups were compared with t test or Kruskal‒Wallis rank sum test. Categorical variables are presented as frequencies, and the chi-square test was used to assess group differences. The LASSO algorithm was used in combination with the ‘glmnet’ package for analysis. Based on the predictive features, a nomogram was established. The goodness-of-fit of this nomogram was tested by the Hosmer–Lemeshow test. Receiver operating characteristic (ROC) curve analysis was used to evaluate the predictive value of this model, and the nomogram’s clinical usefulness was evaluated using calibration and decision curve analysis (DCA). All above statistical analyses were performed using R version 4.1.3 (https://www.r-project.org/). The original dataset was corrected by using Stata 17.0 with 200 bootstrap samples.

## Results

### Patient characteristics

From July 2017 to December 2021, 95 women who had previously undergone tubal sterilization and underwent laparoscopic bilateral tubal anastomosis were selected. All patients’ clinical information was analyzed before modeling. No significant differences were found in BMI, race, education, sterilization duration, FT3, FT4, CA125, HCY, D-dimer, AFC, anastomosis site, hydrosalpinx, hysteromyoma, ovarian cyst, pelvic adhesion or endometriosis. However, patients with lower age or without intrauterine polyps had a higher pregnancy rate (Table 1).

**Table 1:** Patients baseline clinical features analysis

Variables	Total (n = 95)	Non-pregnancy (n = 33)	Pregnancy (n = 62)	p
Age(year)	35.3 ± 4.9	36.9 ± 5.6	34.4 ± 4.4	0.034^*^
BMI(kg/m^2^)	22.6 (21.2, 25.3)	23.2 (21.6, 25.9)	22.3 (21, 25)	0.284
Race				0.415
Han Chinese (%)	89 (93.7%)	30 (90.9%)	59 (95.2%)	
Other ethnic (%)	6 (6.3%)	3 (9.1%)	3 (4.8%)	
Education				0.493
Middle school and blow (%)	77 (81.1%)	25 (75.8%)	52 (83.9%)	
High school and above (%)	18 (18.9%)	8 (24.2%)	10 (16.1%)	
Sterilization duration(years)	9.5 ± 3.9	9.9 ± 4.7	9.3 ± 3.5	0.511
FT3(pmol/L)	5 ± 0.5	5 ± 0.5	5 ± 0.6	0.908
FT4(pmol/L)	14.9 ± 2.1	15.2 ± 2.2	14.8 ± 2.1	0.375
TSH(uIU/mL)	2.1 (1.5, 2.8)	2.3 (2, 3.1)	2 (1.4, 2.7)	0.089
CA125(U/ml)	13.9 (10.8, 15.9)	13.4 (10.8, 15.9)	14.2 (10.8, 15.9)	0.688
HCY(umol/L)	12.3 (10, 14)	12.3 (10, 13.9)	12.4 (10.1, 14.3)	0.684
D-dimer(ug/ml)	0.2 (0.1, 0.3)	0.2 (0.2, 0.3)	0.2 (0.1, 0.3)	0.362
AFC	12 (9, 18)	12 (8, 17)	12 (10, 18)	0.507
Anastomosis site(L)				0.259
isthmus-isthmus (%)	29 (30.5%)	13 (39.4%)	16 (25.8%)	
isthmus-ampulla (%)	45 (47.4%)	12 (36.4%)	33 (53.2%)	
ampulla-ampulla (%)	21 (22.1%)	8 (24.2%)	13 (21%)	
Anastomosis site(R)				0.435
isthmus-isthmus (%)	25 (26.3%)	10 (30.3%)	15 (24.2%)	
isthmus-ampulla (%)	46 (48.4%)	13 (39.4%)	33 (53.2%)	
ampulla-ampulla (%)	24 (25.3%)	10 (30.3%)	14 (22.6%)	
Hydrosalpinx				1
no (%)	91 (95.8%)	32 (97%)	59 (95.2%)	
yes (%)	4 (4.2%)	1 (3%)	3 (4.8%)	
hysteromyoma				0.506
no (%)	84 (88.4%)	28 (84.8%)	56 (90.3%)	
yes (%)	11 (11.6%)	5 (15.2%)	6 (9.7%)	
ovarian cyst				0.655
no (%)	90 (94.7%)	32 (97%)	58 (93.5%)	
yes (%)	5 (5.3%)	1 (3%)	4 (6.5%)	
Pelvic adhesion				0.156
no (%)	54 (56.8%)	15 (45.5%)	39 (62.9%)	
yes (%)	41 (43.2%)	18 (54.5%)	23 (37.1%)	
Intrauterine polyps				0.01^*^
no (%)	82 (86.3%)	24 (72.7%)	58 (93.5%)	
yes (%)	13 (13.7%)	9 (27.3%)	4 (6.5%)	
Endometriosis				0.484
no (%)	55 (57.9%)	17 (51.5%)	38 (61.3%)	
yes (%)	40 (42.1%)	16 (48.5%)	24 (38.7%)	

Continuous variables are expressed in mean ± standard deviation (SD) or median (25th–75th percentiles). Categorical variables were expressed as frequencies (percentages). ^*^P value<0.05

**Abbreviations**: FT3: Free Triiodothyronine, FT4: free thyroxine, TSH: Thyroid-stimulating hormone, CA125: Carbohydrate antigen 125, HCY: homocysteine, AFC: Antral Follicle Count

### Machine learning model by LASSO

**Fig. 1:**
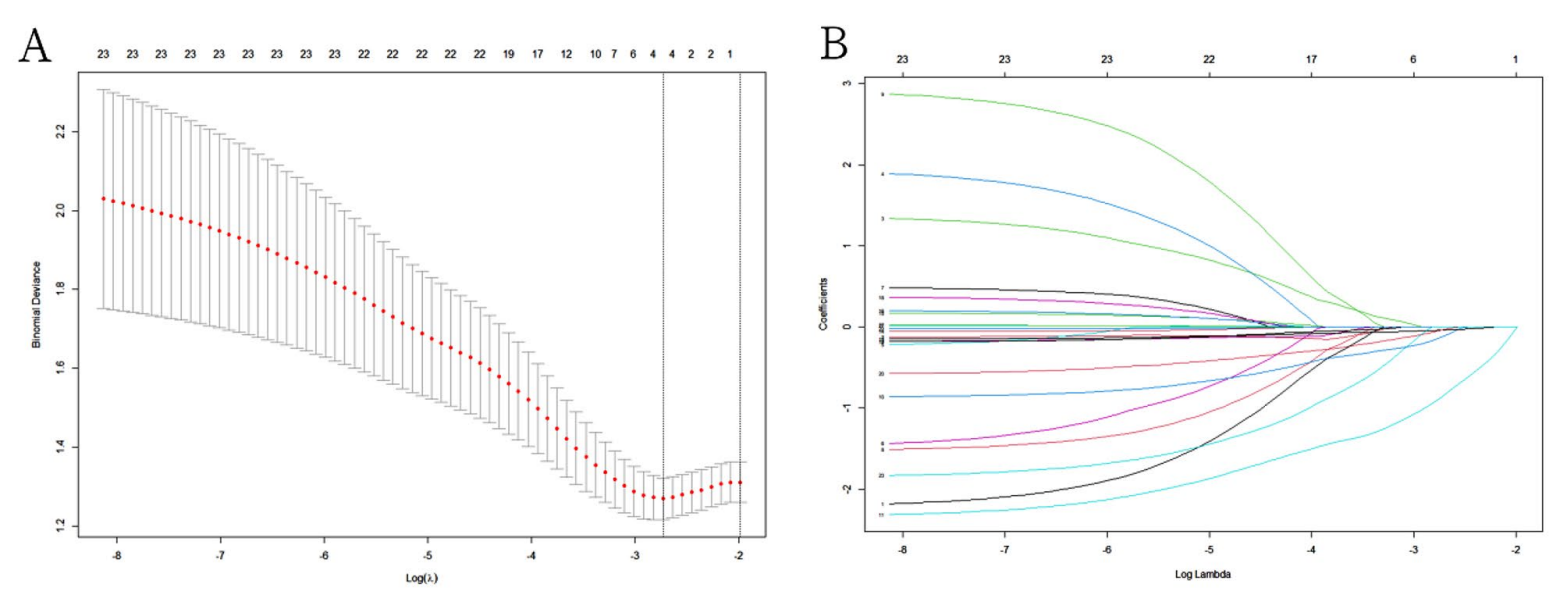
Clinical feature selection using the least absolute shrinkage and selection operator (LASSO) binary logistic regression model. **(A)** (Tuning parameter (λ) selection in the LASSO model used 10-fold cross-validation via minimum criteria. The area under the receiver operating characteristic (AUC) curve was plotted versus log(λ). Dotted vertical lines were drawn at the optimal values by using the minimum criteria and the 1 standard error of the minimum criteria the 1-SE criteria. **(B)** LASSO coefficient profiles of the 19 clinical features. A coefficient profile plot was produced against the log (λ) sequence

All parameters were analyzed as shown in the LASSO algorithm, and the model was finally built (Fig. 1). Four elements, including age, intrauterine polyps, pelvic adhesion and TSH, were selected as the best subset of factors to develop the clinical pregnancy prediction model. The clinical pregnancy prediction model calculation formula was as follows: clinical pregnancy prediction model score = -0.037*age-0.124*pelvic adhesion − 0.863*intrauterine polyps − 0.041*TSH + 2.230.

### Development of a predictive nomogram model in women after LTA

**Fig. 2:**
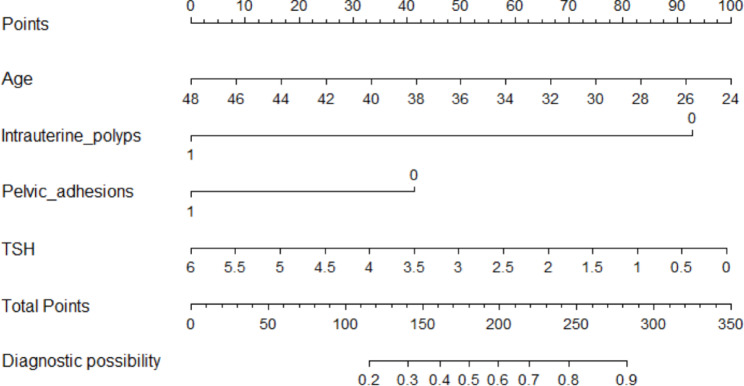
Developed clinical pregnancy nomogram. The model was developed in the original dataset, with age, Intrauterine polyps, Pelvic adhesion and TSH incorporated

**Fig. 3:**
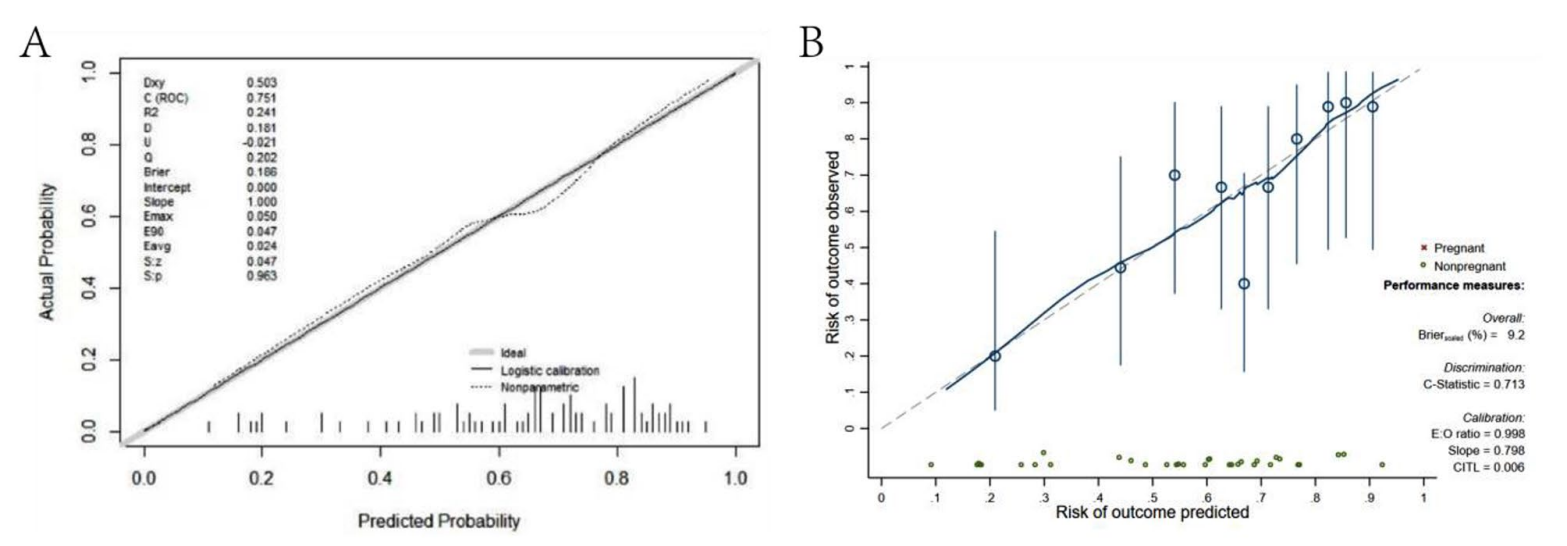
Calibration curves of the clinical pregnancy nomogram **(A)**. Calibration curves was corrected with 200 bootstrap samples **(B)**

**Fig. 4:**
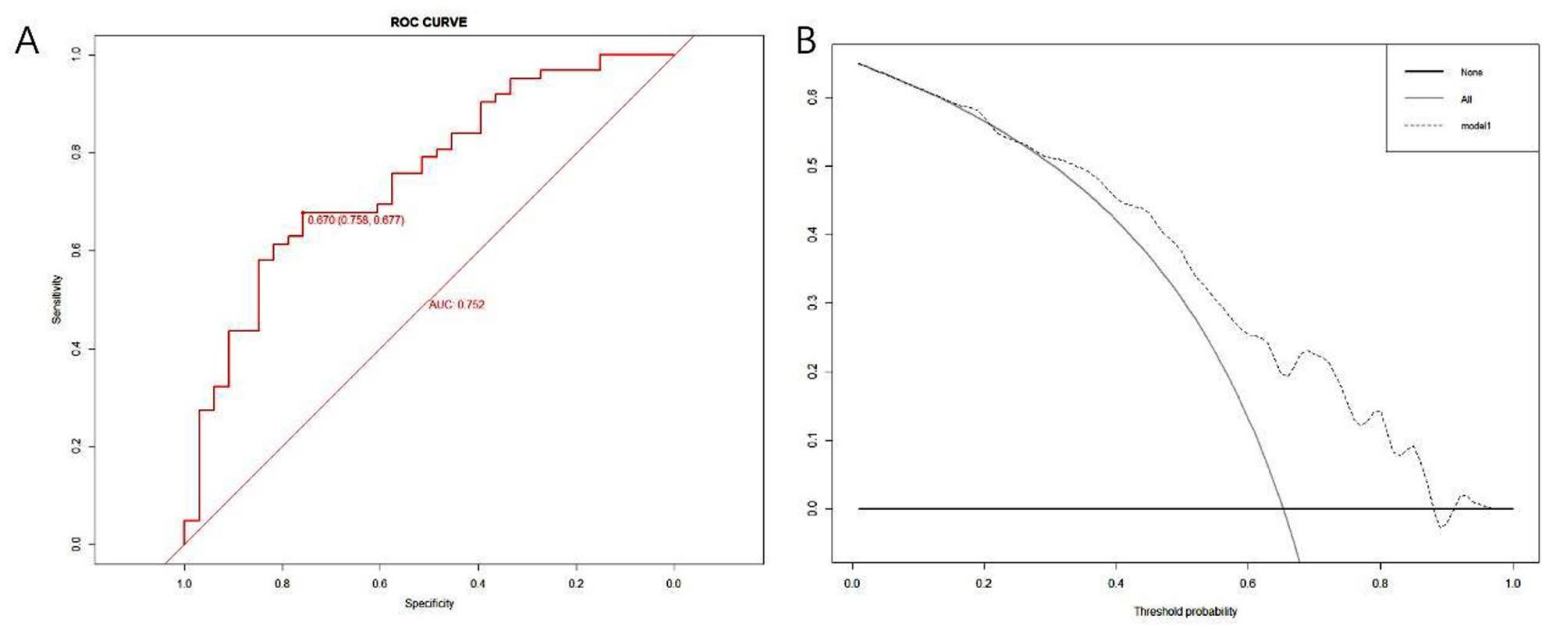
ROC curve analyses compare the predictive performance **(A)**. DCA for the clinical pregnancy nomogram **(B)**

A nomogram with the abovementioned four predictive factors was created to institutionally show the prediction model (Fig. 2). A total score was obtained by adding matching points for each parameter in the nomogram to evaluate clinical pregnancy possibility. The Hosmer‒Lemeshow test calibration showed that χ^2^ was 4.955 and the p value was 0.838, indicating a satisfactory goodness-of-fit. Figure 3 A shows the calibration curve, which suggested that the clinical pregnancy indicated by the nomogram was essentially accurate. Discriminatory capacity and generalizability were analyzed using ROC curves, and the AUC of the original data results was 0.752 (Fig. 4A). DCA is shown in Fig. 4B, which indicates that the model performed well and was feasible for making beneficial clinical decisions. Internal validation shows the performance indices of the model corrected for optimism after 200 bootstrap samples (Fig. 3B). Overall, the predictive model performs well even after optimistic correction.

## Discussion

The pregnancy outcome after LTA is affected by multiple factors, and the probability of successful treatment is of great concern to patients. A systematic review including 37 studies concluded that the pooled pregnancy rate following sterilization reversal was 42–69%, and the reported ectopic pregnancy rate was 4–8% [13]. Currently, physicians only make rough estimates and cannot individualize information on the probability of successful pregnancy after LTA treatment. As a result, patients missed valuable treatment opportunities due to uncertainty about whether or not to continue to try natural pregnancy. Therefore, in the process of receiving patients by clinicians, individualized prediction can provide objective data in combination with the patient’s own indicators, and it is necessary to have reasonable expectations for the pregnancy outcome.

Many factors affect pregnancy outcome after LTA. Different studies included different variables, and the conclusions of the studies were not consistent. A previous study including 156 cases of LTA concluded that age, time of ligation, anastomosis site and tubal length were associated with pregnancy rates in laparoscopic tubal recanalization [14]. According to a study by Xavier DeYeux, age, the type of ligature, the anastomosis site, the length of remaining tube, and the years of ligation all determine whether tubal patency can be restored [15]. A retrospective study including 127 women studied whether clinical characteristics could affect the pregnancy outcome and concluded that only age affects the probability of positive pregnancy [7]. To identify factors influencing pregnancy outcomes after LTA, we collected baseline data, preoperative tests and laboratory parameters of patients undergoing LTA and innovatively included intraoperative conditions as study variables. After machine learning, LASSO regression identified age pelvic adhesions, endometrial polyps and TSH as independent risk factors for clinical pregnancy after LTA. However, we did not find a significant difference in the site of anastomosis or sterilization duration.

In this study, we developed a clinical model containing age, intrauterine polyps, pelvic adhesion and TSH to help evaluate the probability of clinical pregnancy in women after receiving LTA. The prediction model showed good calibration and discrimination, with an area under the ROC curve of 0.752. Age is a strong prognostic factor affecting the possibility of conception after reversal of female sterilization. Our findings are consistent with previous studies. According to a prior study, IVF was the most cost-effective approach for the oldest women (over 41 years of age) who wanted to have children following tubal ligation, while tubal anastomosis was the most cost-effective method for the majority of women under the age of 41 [16]. It is generally believed that for women over 40 years of age, oocyte quality gradually deteriorates and is accompanied by a reduced pregnancy rate [17, 18]. In our study, patients in the pregnancy group were younger, and the difference was statistically significant. Our study also found that patients with pelvic adhesion may have a lower pregnancy rate. The term pelvic adhesions refers to tubal adhesions, ovarian adhesions, and oviduct adhesions caused by inflammation in pelvic tissues [19]. Pelvic adhesions can impair the structure and function of the fallopian tubes, leading to infertility [20]. In addition, we discovered that patients with endometrial polyps had a decreased pregnancy rate. Endometrial polyps are common in infertile women, and the prevalence rate is as high as 32% [21]. In fact, endometrial polyps are more likely to exhibit aberrant molecular expression, which hinders implantation and early embryonic development [22]. Endometrial polyps may negatively impact fertility through various pathways, including mechanical interference and the production of chemicals that interfere with sperm transport or embryo implantation [23]. Evidence reveals that endometrial receptivity is significantly impacted by elevated levels of aromatase and lower levels of HOXA-10 and HOXA-11 mRNA [24, 25]. In addition, we found that TSH is also a key factor affecting the pregnancy rate after surgery. Normal thyroid function is important to maintain normal reproduction [26, 27]. Thyroid hormone and hormone receptors also play a role in regulating endometrial receptivity. Additionally, modifications in thyroid hormone transmission may harm the placenta and potentially result in miscarriage [28, 29].

In summary, we present a nomogram incorporating age, intrauterine polyps, pelvic adhesion and TSH that can be conveniently used to predict clinical pregnancy in women after LTA. Intraoperative tubal anastomosis combined with TSH and age can help to evaluate the clinical pregnancy more accurately.

This study has several advantages. First, by incorporating intraoperative conditions as research variables improved the reliability of our research. Second, the same surgeon operated on each patient, eliminating any potential impact of the surgeon’s skill level on the success of the pregnancy. However, our study also had certain limitations. First, in this study, we solely focused on the outcome of pregnancy without considering the time interval between surgery and conception, as well as potential infertility factors like ovulatory dysfunction. However, these factors could potentially be confounding factors that affects reproductive outcomes. Therefore, future studies should take this aspect into account when examining the relationship between surgery and reproductive outcomes. Secondly, indicators that are essential for assessing ovarian reserve, such as basal follicle-stimulating hormone (FSH) and anti-Mullerian hormone (AMH) levels, are crucial factors affecting postoperative pregnancy. However, as most participants in this study were not tested for these indicators, they were not incorporated in the model. Future studies should consider including these variables. Third, the statistical power was restricted by the relatively small sample size of just 95 patients in this retrospective analysis. To verify our results, a multicenter prospective trial is needed.

## Data Availability

The used data and materials during the present study are available from the corresponding author on reasonable request.
